# Inoculation of Saliva

**Published:** 1881-11

**Authors:** 


### INOCULATION OF SALIVA.

M. Vulpian injected under the skin of rabbits saliva collected at the very moment of the experiment from perfectly healthy individuals; and this injection killed the rabbits so inoculated in forty-eight hours. The blood of these rabbits was found filled with microscopic organisms; among which was found a special organism, discovered by M. Pasteur in the course of his experiments with inoculation of the saliva of a child who had died of rabies. One drop of this blood, diluted in ten grammes of distilled water, and injected under the skin of other rabbits, also brought on the death of these animals: the blood of which was similarly filled with microscopic organisms. Rabbits placed in identical conditions, and inoculated with the same saliva, experienced no ill effects from their inoculation, and continued in excellent health.—*British Medical Journal.*

